# Oxidonitrergic and antioxidant effects of a low molecular weight peptide fraction from hardened bean (*Phaseolus vulgaris*) on endothelium

**DOI:** 10.1590/1414-431X202010423

**Published:** 2021-04-19

**Authors:** D. Graziani, J.V.V. Ribeiro, V.S. Cruz, R.M. Gomes, E.G. Araújo, A.C.M. Santos, H.C.M. Tomaz, C.H. Castro, W. Fontes, K.A. Batista, K.F. Fernandes, C.H. Xavier

**Affiliations:** 1 Laboratório de Neurobiologia de Sistemas, Instituto de Ciências Biológicas, Universidade Federal de Goiás, Goiânia, GO, Brasil; 2 Laboratório Multiusuário de Avaliação de Moléculas, Células e Tecidos, Escola de Veterinária e Zootecnia, Universidade Federal de Goiás, Goiânia, GO, Brasil; 3 Laboratório de Bioquímica e Química de Proteínas, Departamento de Biologia Celular, Universidade de Brasília, Brasília, Brasil; 4 Laboratório de Química de Polímeros, Instituto de Ciências Biológicas, Universidade Federal de Goiás, Goiânia, GO, Brasil; 5 Instituto Federal de Educação, Ciência e Tecnologia de Goiás - Campus Goiânia Oeste, Goiânia, GO, Brasil; 6 Laboratório de Fisiopatologia Cardiovascular e Neurológica, Instituto de Ciências Biológicas, Universidade Federal de Goiás, Goiânia, GO, Brasil

**Keywords:** Phaseolus vulgaris, Hardened bean, Peptide, Oxidative stress, Nitric oxide

## Abstract

About 3000 tons of beans are not used in human food due to hardening. Several studies on bean-derived bioactive peptides have shown potential to treat some diseases, including those relying on oxidative dysfunctions. We assessed the effects of peptides extracted from hardened bean *Phaseolus vulgaris* (PV) on reactive oxygen species (ROS) and nitric oxide (NO) production, cytotoxic and cytoprotective effects in endothelial cells, and oxidonitrergic-dependent vasodilating effects. Extract was composed by peptide fraction <3 kDa (PV3) from hardened common bean residue. PV3 sequences were obtained and analyzed with bioinformatics. Human umbilical vein endothelial cells were treated with 10, 20, 30, and 250 µg/mL PV3. Oxidative stress was provoked by 3% H_2_O_2_. Cytotoxicity and cytoprotective effects were evaluated by MTT assay, whereas, ROS and NO were quantified using DHE and DAF-FM fluorescent probes by confocal microscopy. NO- and endothelium-dependent vasodilating effects of PV3 were assessed in isolated aortic rings. We found 35 peptides with an average mass of 1.14 kDa. There were no cell deaths with 10 and 20 μg/mL PV3. PV3 at 30 μg/mL increased cell viability, while cytotoxicity was observed only with 250 μg/mL PV3. PV3 at 10 μg/mL was able to protect cells from oxidative stress. PV3 also increased NO release without causing cell death. It also reduced relative ROS production induced by H_2_O_2_. PV3 vasodilating effects relied on endothelium-dependent NO release. PV3 obtained from low-commercial-value bean displays little cytotoxicity and exerts antioxidant effects, whereas it increases endothelial NO release.

## Introduction

According to the global consumption ranking, *Phaseolus vulgaris* (PV) is the third most important legume, preceded only by soybean (*Glycine max*) and peanut (*Arachis hypogea*). For millions of people across the world, beans are the major source of protein, dietary fiber, iron, complex carbohydrates, minerals, and vitamins (1). Bean storage should be a careful procedure, since grain cells are still alive, and their metabolic processes remain active. Increases in the time required for cooking due to excessive hardening of the grain may be a consequence of damage resulting from inadequate storage, such as: i) shell cracking due to environmental low humidity; ii) pests and fungi; iii) bark darkening that reflects morphological changes, commonly found in lighter cultivars; and iv) grain weight loss (2). These morphological, chemical, and physical changes result in a condition known as the hard-to-cook (HTC) effect.

Insolubilization of pectic substances by the phytase enzyme is a widely accepted mechanistic hypothesis (3); however, there are other enzymatic reactions that may be involved in the hardening process. Among these additional hypotheses, the following can be highlighted: formation of insoluble pectates; increase in the content of condensed tannins in the husk and migration of soluble tannins to the grain cotyledons; complex formation between tannins and medium lamella macromolecules; denaturation and/or protein association; complex formation between polyphenols, proteins, and pectins; cell wall lignification; oxidation and/or lipid polymerization (4). These effects can be accelerated when storage conditions have high air humidity (>75%) and high temperatures (30-40°C), which are characteristics of tropical countries (5). Therefore, HTC beans are usually disposed of and as a consequence, there is a large amount of non-commercially viable residues. However, this amount of disposable beans may still be used as a source of nutraceutical components (6,7), thus avoiding food wasting and obtaining some therapeutic benefits.

Several biological effects of protein extracts produced from beans have been described, such as: antihyperglycemic (7), anticancer (8), ACE inhibitory and potentially antihypertensive (9), anti-inflammatory (10), and antioxidant (11). Notwithstanding the aforementioned results obtained in non-HTC, it is worth hypothesizing that HTC beans may also serve as a source of bioactive compounds. In this study, we purified a low molecular weight peptide fraction (<3 kDa) from HTC *Phaseolus vulgaris* (PV3) and assessed the potential oxidonitrergic and antioxidant effects of this fraction in endothelial cells and in isolated vessels.

## Material and Methods

### PV3 extract

The bean grains were supplied by the Brazilian Agricultural Research Corporation (EMBRAPA) Rice and Beans (Brazil), stored in a greenhouse for 120 days at 40°C (relative humidity of 75%). Flour was produced from hand-peeled beans, passed through a knife mill, and then sieved. The flour was stored in sealed plastic bags and refrigerated at -20°C for further use.

The peptide fraction (<3 kDa) was from *Phaseolus vulgaris* Pontal cultivar hardened common bean residue, from the carioca commercial group. For peptide extraction, 5 mL of a solution containing acetonitrile, water, and formic acid (ratio of 25:24:1) was added to every 1 g of flour. The sample was stirred for 1 h at room temperature and centrifuged at 11,200 *g* for 10 min at room temperature. The supernatant underwent ultrafiltration on a 3 kDa membrane (Amicon Bioseparations, Germany). Then, the extract was lyophilized and kept at 8°C. For each test, the extract was diluted in saline (0.9% NaCl). The filtrate obtained (peptides smaller than 3 kDa) in the process was denominated PV3. The amount of protein in the PV3 was determined by the Qubit^®^ Protein Assay kit and Qubit^®^ Fluorometer (Invitrogen, USA).

### PV3 peptidomics by mass spectrometry coupled liquid chromatography (LC/MS-MS)

The samples were analyzed in a liquid chromatography system (nano-UHPLC Dionex Ultimante 3.000) coupled online to the Orbitrap Elite™ hybrid ion trap-orbitrap mass spectrometer (Thermo Scientific, Germany). Two types of capillary columns were used in the chromatographic system, a 100 µm × 200 mm long internal diameter pre-column packed in the laboratory with 5 µm ReprosilPur 5 µm spherical silica particles with C18 Å (Dr. Maisch, Germany) and a 75 µm × 35 cm long analytical column, also packaged in the laboratory, with 3 µm C18 Reprosil particles with 120 Å pores (Dr. Maisch). Samples were eluted using a gradient of 98% solution A (0.1% formic acid in water) to 35% solution B (0.1% formic acid in ACN) over 155 min, 35 to 98% solution B for 5 min, and 98% solution B for 8 min (a total of 168 min at 230 nL/min). After each run, the column was equilibrated with 98% solution A. Mass spectra were acquired in positive mode by applying data-dependent automatic survey MS scan and tandem mass spectral acquisition (MS/MS). All MS scans in the orbitrap (mass range: m/z 350-1650, resolution: 120,000) were followed by MS/MS of the fifteen most intense ions. Fragmentation by collision-induced high energy dissociation (HCD) and repeated ion fragmentation were dynamically prevented.

### Computational analysis

Peaks v.7.0 software (BSI Bioinformatics Solutions Inc., Canada) was used to perform *de novo* sequencing and peptide spectrum matches (PSM) search. The search was performed against a database containing the proteins found in the UniProt repository (http://www.uniprot.org), filtered by *Phaseolus vulgaris* taxonomy ID (3885). Redundancies were removed. The searches were performed with the following parameters: 10 ppm MS tolerance, 0.05 Da MS/MS tolerance, no cleavage agent was selected (since the goal of this study was to analyze naturally occurring peptides), methionine oxidation was selected as variable modification. The number of proteins, the protein group, and the number of peptides were filtered with a false positive detection rate (FDR) of less than 1% and peptides with rank 1 and minimum of 2 peptides per protein were accepted for identification.

The sequenced peptides were analyzed following the method of Mojica et al. (12). Sequence confirmation was performed using BLAST^®^(https://blast.ncbi.nlm.nih.gov/Blast.cgi, as accessed in February 27, 2020). Only peptide sequences that showed alignment with the bean genome were maintained in order to eliminate contaminants. The biological activity of the peptides was predicted using the BIOPEP database (http://www.uwm.edu.pl/biochemia, as accessed in February 27, 2020) (13).

### Antioxidant effect of PV3 by DPPH assay

Antioxidant activity was determined using DPPH (2,2-diphenyl-1-picrylhydrazyl) as an antioxidant agent. Two hundred microliters of DPPH (0.15 mM) and 50 µL of sample (concentrations ranging from 0.1 to 0.5 mg/mL) were used. Following 15 min, samples were analyzed in a microplate spectrophotometer (EPOCH) at a wavelength of 520 nm. Determination of antioxidant activity was expressed through the calibration curve using Trolox (R^2^=0.9956) (14).

### Effect of PV3 on human endothelial cells

Human umbilical vein endothelial cells were purchased from the Rio de Janeiro Cell Bank (UFRJ, Brazil) originated from the ATCC (American Type Culture Collection, USA). Cultivation was performed in a humidified incubator at 37°C and 5% CO_2_ in bottles using modified Eagle's Dulbecco's medium (DMEM) plus 10% fetal bovine serum, amphotericin B, and L glutamine (Cultilab, Brazil).

After the cultivation step, the cells were quantified in a Neubauer chamber and seeded onto 96-well plates containing 200 μL DMEM medium at 1×10^4^ concentrations. Then, the medium was discarded, and the wells containing cells were treated with PV3 at concentrations of 10, 20, 30, and 250 µg/mL for 24 h. The negative control group was treated with extract diluent, 0.9% (w/v) sodium chloride solution. The positive control group was treated with diluent and, after 24 h, was exposed to 3% (v/v) hydrogen peroxide (H_2_O_2_) for one hour. All assays were performed in three independent and triplicated experiments.

#### Effect of PV3 on cytotoxicity and cell viability assay by tetrazolium reduction (MTT) assay

After the treatment period, 10 µL of MTT [3-(4,5-dimethyl-2-thiazolyl)-2,5-diphnyl-2H-tetrazolium], previously diluted in phosphate buffered saline (PBS, in the ratio of 500 mg MTT to 100 mL PBS), was added to the plates and kept in a humidified incubator for 3 h. Subsequently, 50 µL of 10% sodium dodecyl sulfate (SDS, Vivantis Biochemical, Malaysia) was added to stop the reaction. The plates were kept at room temperature for 24 h and protected from light.

The absorbance of the wells was quantified by spectrophotometry (425-540 nm, Stat Fax 2100, Awareness Technology Inc., USA). Graph production and statistical tests were carried out using the GraphPad Prism program (GraphPad Software, USA). Cytotoxicity (CT) was estimated using the equation: %CT = 100 - (absorbance under treatment/absorbance under control condition) × 100.

To evaluate the cytoprotective potential of PV3 against oxidative stress, after the cell treatment period, the supernatant was discarded and a new medium containing 3% (v/v) H_2_O_2_ was added. After one hour of exposure to the H_2_O_2_ stressor, the plates were washed twice with PBS buffer and tetrazolium reduction analysis was performed (15). The specimens were observed with confocal microscopy (TCS SP8 DMi8, Germany) on a 10× objective by Normarski interferential differential contrast microscopy (DIC) to obtain images of cells before and after treatment. Cell viability (CV) was estimated by the equation: %CV = (absorbance under treatment / absorbance under control condition) × 100

#### Effect of PV3 on nitric oxide and free radical production

This protocol was similar to previously described (16). To evaluate whether PV3 treatment was able to induce nitric oxide (NO) production, the cell confluents on a polarized slide were incubated for 20 min at 37°C with 1 µM DAF-FM™ (Sigma-Aldrich, Germany). Then, the slide was washed with PBS to remove excess probe. After 20 min, cell images were taken with confocal microscopy (TCS SP8 DMi8) on 10 and 40× objective lenses. The product generated by the reaction with the DAF-FM probe was excited with the argon laser line at 488 nm and the fluorescence emission intensity measured at 515 nm. The product generated by the reaction with the DAPI probe was excited with the Diode 405 laser line at 410 nn with the fluorescence emission intensity measured at 360 nn. Images from 10 different fields were captured in which DAPI (fluorescent probe for DNA labelling) and DAF-FM (fluorescent probe for NO detection) were analyzed, generating three image markings: DNA, NO, and DNA and NO merging.

To evaluate reactive oxygen species (ROS), after treatment, the cell confluents on polarized slides were incubated for 20 min at 37°C with 10 µM DHE™ probe (Thermo Fisher Scientific, EUA). Then, the slides were washed with PBS to remove excess probe. After 20 min, the product generated by the reaction with the DHE probe was excited with the argon laser line at 496 nm and the fluorescence emission intensity measured at 550 nm. DIC microscopy was also used to obtain cell images without revealing probe labelling. Images from 10 different fields were captured in which the DHE labeling (fluorescent probe for reactive oxygen species) was analyzed, thus generating three images: ROS labeling, cells in DIC, and image merging. This protocol was similar to that previously described (17).

For quantitative analysis, Image J software (NIH, USA) was used to enable the analysis of the pixels generated from the specific excitation evoked from cell incubation with DAF-FM and DHE. The amount of NO and ROS was quantified as an average percentage of fluorescence pixels detected in ten areas of 50 square micrometers.

### Isolated aortic ring preparation

All experiments were approved by the local institutional animal welfare committee at UFG (CEUA/UFG, protocol number 084/2018) and were conducted in accordance with the Brazilian Federal Law No. 11.794. We made all efforts to minimize the number of animals used. The rats were maintained in controlled temperature (22±2°C) with a 12-h light/dark cycle and had free access to water and food. Isolated aortic rings from male Wistar rats (250-300 g) were used to evaluate the vascular effects of PV3, as follows.

Following decapitation, the descending thoracic aorta was removed and sectioned into 4-mm rings. The aortic rings were placed in 10 mL of organ bath at 37°C containing gassed (95% O_2_ and 5% CO_2_) Krebs-Henseleit solution (KHS) with the following composition: NaCl (118.06 mM), KCl (4.6 mM), NaHCO_3_ (24.9 mM), MgSO_4_·7H_2_O (2.4 mM), CaCl_2_·2H_2_O (3.3 mM), KH_2_PO_4_ (0.9 mM), and glucose (11.1 mM), pH 7.4. The rings were fixed to an isometric force transducer under a tension of 1.5 g for 1 h for stabilization. The mechanical activity was recorded using a data acquisition system (DATAQ Instruments, USA). The vasorelaxant effect of PV3 was investigated in rings with intact (E+) or denuded (E-) endothelium. Endothelial integrity was assessed by the degree of relaxation induced by acetylcholine (ACh, 10^-5^ mol/L) in rings pre-contracted with 10^-7^ mol/L phenylephrine (Phe). Functional endothelium was considered when Ach-induced relaxation was greater than 80%. In denuded endothelium rings, the absence of functional endothelium was verified by the absence of Ach-induced relaxation rings pre-contracted with Phe. After an equilibration period (30 min), the aortic rings were pre-constricted with Phe (10^-7^ mol/L), and PV3 (10^-7^, 10^-6^, or 10^-5^ g/L) was added to the KHS. In some experiments, the rings were pre-incubated with the L-NAME (10^-6^ mol/L), a nitric oxide synthase (NOS) inhibitor.

### Statistical analysis

The data are reported as means±SE. Comparisons were analyzed by two-way ANOVA, followed by Fisher (for experiments in cells) and Sidak (for experiments in isolated vessels) *post hoc* tests. All analyses were performed using GraphPad Prism 6.0 software. The level of significance was set at P<0.05. The cell culture tests were performed in at least three independent replications and the analyses were performed by comparing averages of triplicates.

## Results

Protein quantification of the extract revealed the presence of 120.4±30.8 mg/g. Based on the UniProt database, it was possible to identify 35 peptides present in the PV3 extract that corresponded to 20 proteins from *Phaseolus vulgaris*. The average length of the peptides identified was 10.2 amino acids and average mass equal to 1.14 kDa. The largest characterized peptide, PV3-A09, has 23 amino acids in its composition and a mass of 2.35 KDa. The smallest peptides have 5 amino acids in their primary structure. Supplementary Table S1 presents the sequences, length, molecular weight, coverage, code of the source protein (Uniprot), source protein length, and biochemical characteristics of the source protein according to Uniprot.

While assessing sequences by the BIOPEP software, it was observed that many peptides had potential for more than one biological activity. All sequences showed potential for inhibiting ACE and DPP IV. The potential for DPP III inhibition was present in 59% of the analyzed sequences. A potential antioxidant predicted effect was found in 41% of the sequences. Other bioactive potentials predicted by the software were: stimulating effect in 29% (for glucose uptake or release vasoactive substance), renin inhibitor in 26%, regulator in 15% (activity of the gastric mucous membrane or ion-flow regulators), mediator of ubiquitin proteolysis activation in 15%, caMPDE inhibitor in 15%, antiamnesia in 9%, antithrombotic in 9%, anti-inflammatory in 9%, and hypolipidemic, neuropeptide, and opioid in 3% for each. These results are detailed in Table 1.

The antioxidant potential of the PV3 peptide fraction was evaluated by the DPPH assay. The PV3 acted as a donor of hydrogen atoms for the oxidoreduction reaction and this resulted in an antioxidant effect, reducing 50% of the DPPH radical at an IC50 of 7.74±0.33.

**Figure 1:**
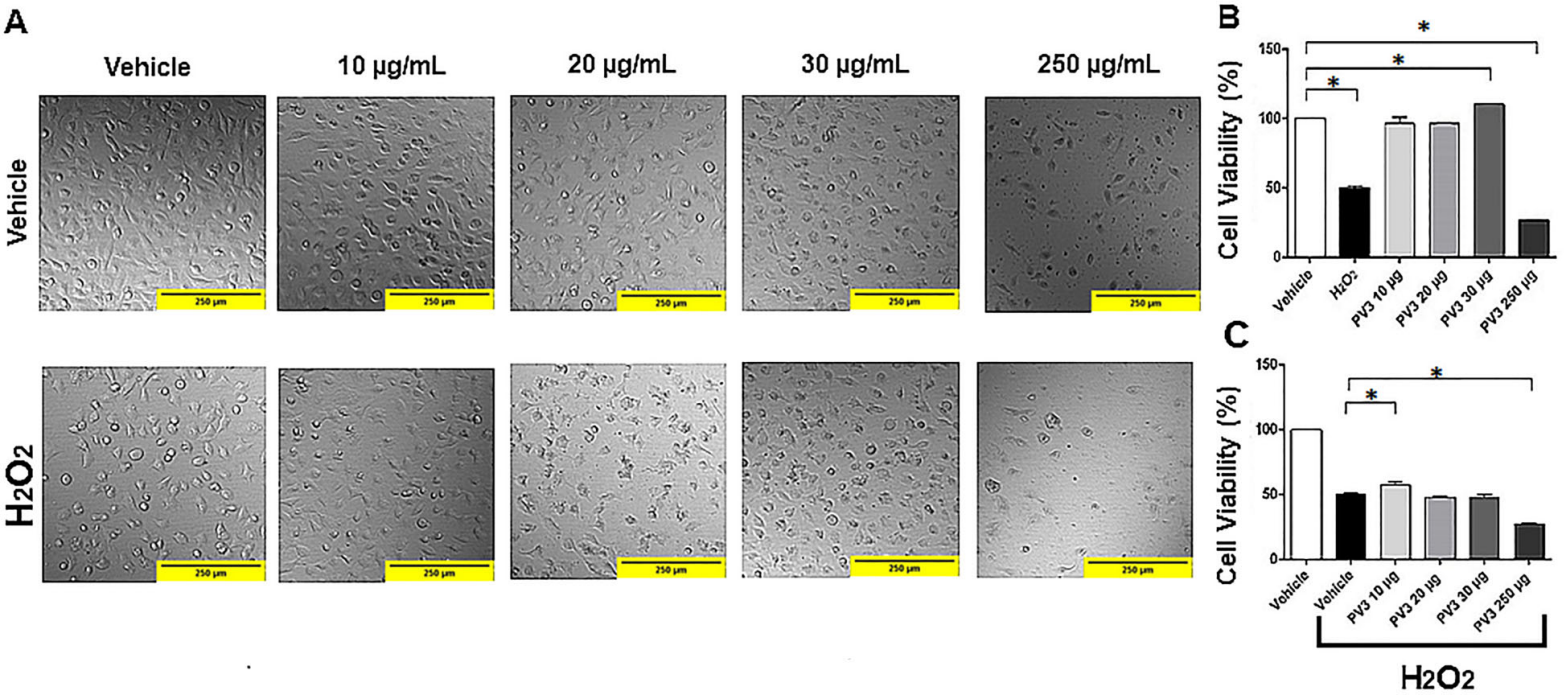
A, Qualitative comparison of *Phaseolus vulgaris* peptide (PV3) treatments at different concentrations in pre-stress and post-oxidative stress by 3% (v/v) H_2_O_2_ for 1 h (100× magnification, scale bar 250 μm, Nomarski interference contrast microscopy). **B**, Evaluation of PV3 cytotoxicity at different concentrations in human endothelial cells by MTT assay. **C**, Evaluation of the cytoprotective potential of PV3 pretreatment against oxidative stress caused by 3% H_2_O_2_ in MTT assay. The data are reported as means±SE. *P<0.05 (ANOVA and Fisher test).

Figure 1 shows the results obtained from the MTT assay, which determines cells viability and death induced by incubation with PV3 at different concentrations and with H_2_O_2_ as a potential cell damager (positive control). DIC microscopy analysis (Figure 1A) was performed to double-check the quantitative findings obtained by the MTT assay. This method allowed demonstrating qualitatively PV3 effects in different concentrations with regard to the possible ability of preventing oxidative stress and of modifying the amplitude of responses from an oxidative challenge.

**Figure 2:**
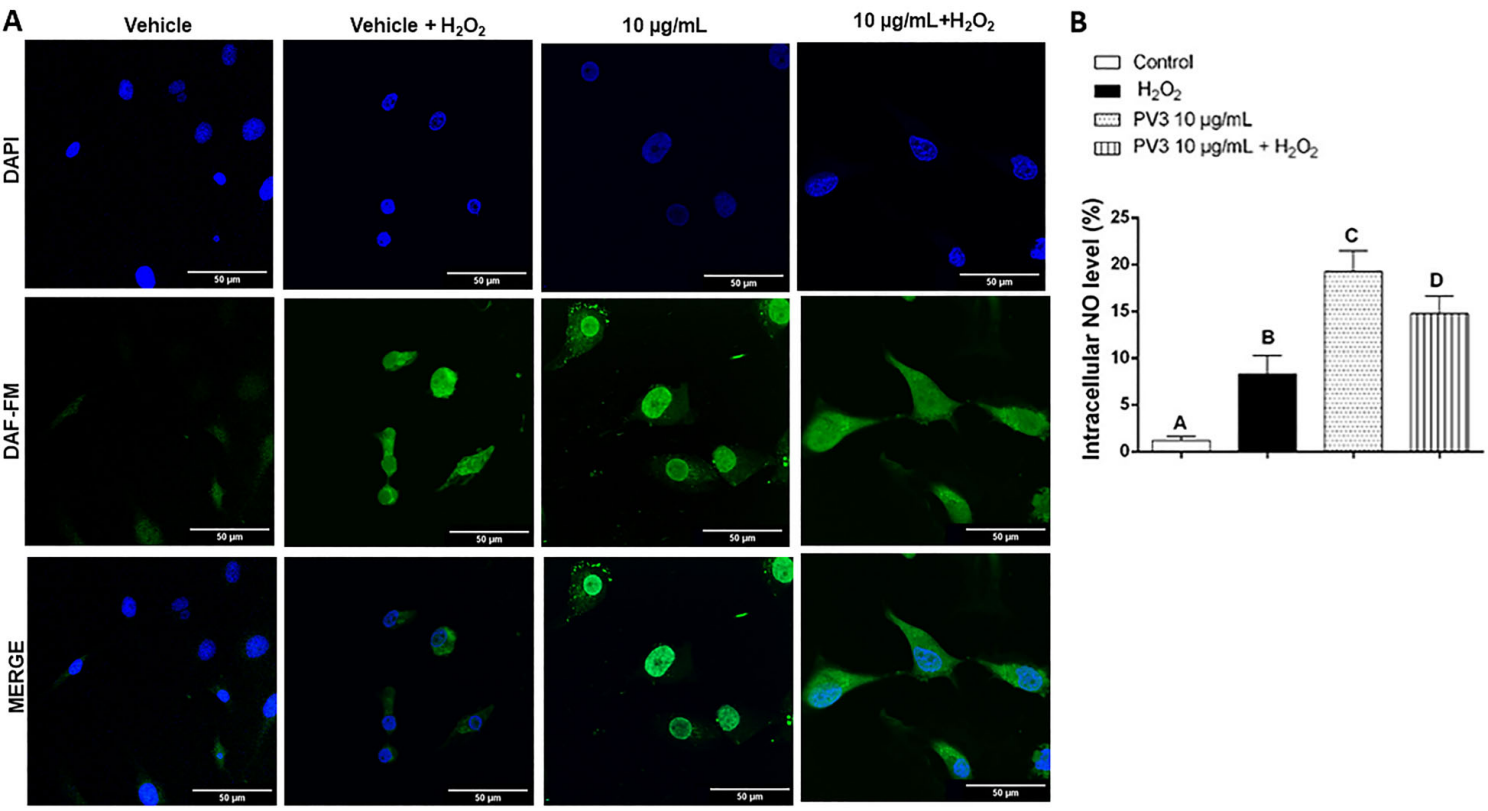
**A**, Effect of *Phaseolus vulgaris* peptide (PV3) extract on nitric oxide (NO) production with and without oxidative stress caused by 3% H_2_O_2_ for 1 h by DAF-FM probe and confocal microscopy (scale bar 50 μm). **B**, Quantification of pixels generated from the specific excitation of NO molecules. The data are reported as means±SE. P<0.05, different upper-case letters indicate that all groups differed from each other (ANOVA and Fisher test).

**Figure 3:**
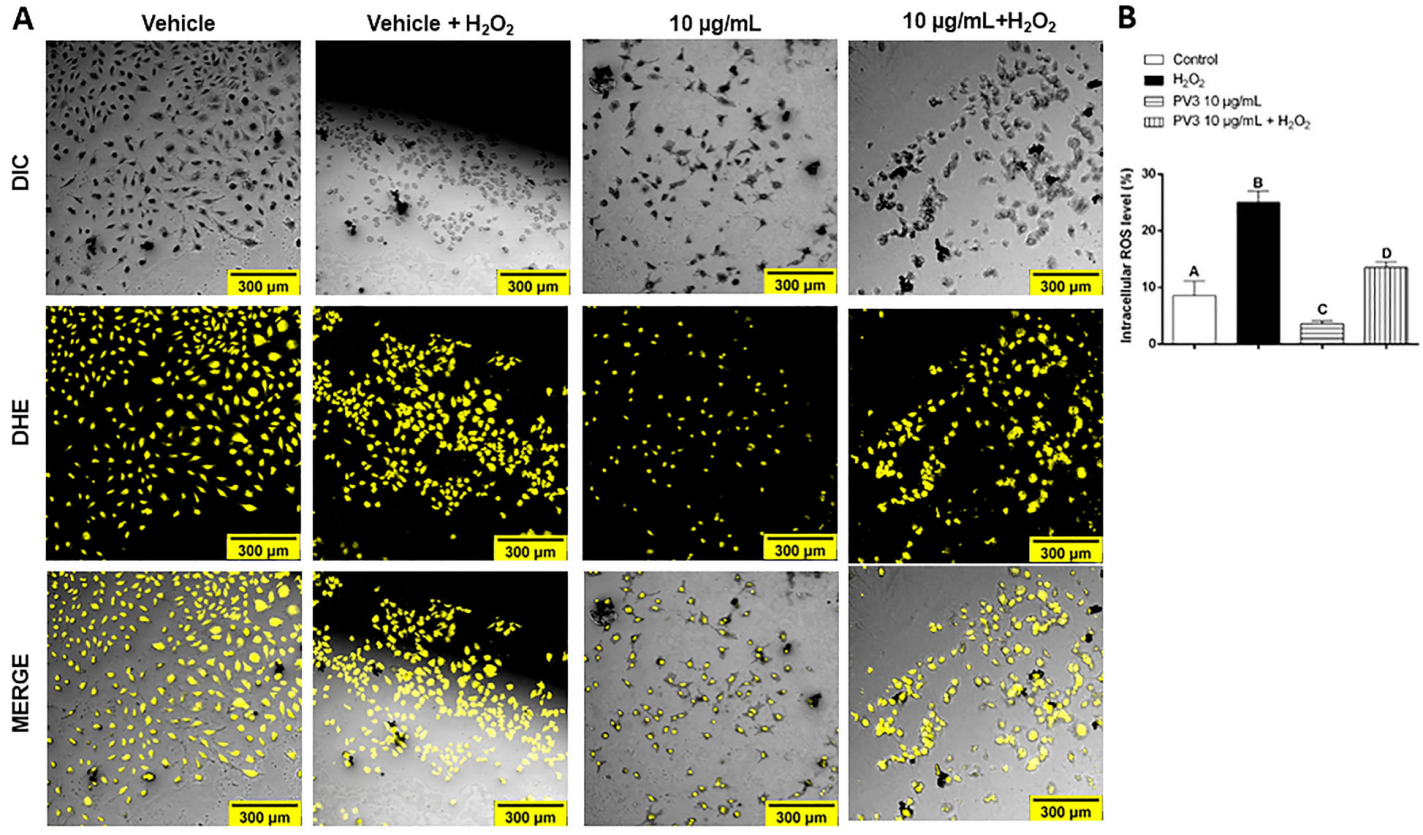
A, Confocal microscopy and DHE probe analysis of the effect of H_2_O_2_ and *Phaseolus vulgaris* peptide (PV3) on reactive oxygen species (ROS) (scale bar 300 μm). **B**, Quantification of pixels generated from the specific excitation of ROS molecules The data are reported as means±SE. P<0.05, different upper-case letters indicate that all groups differed from each other (ANOVA and Fisher test).

**Figure 4:**
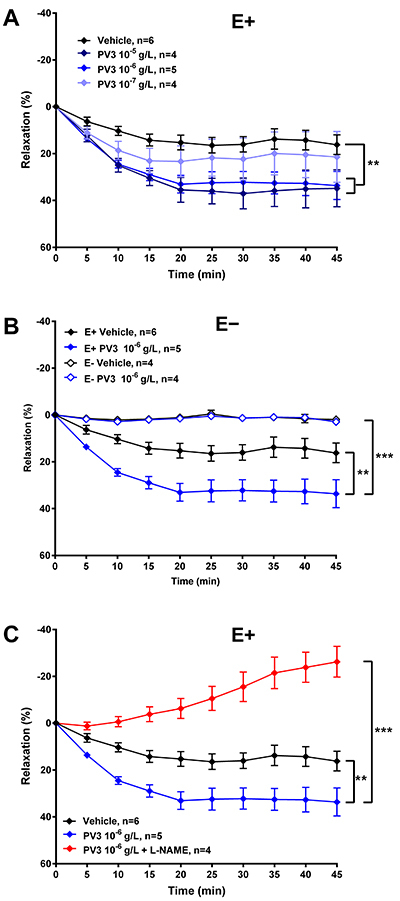
A, Effect of *Phaseolus vulgaris* peptide (PV3) (10^-7^, 10^-6^, or 10^-5^ g/L) on the intact endothelium (E+) aortic rings. **B**, Effects of PV3 (10^-6^ g/L ) in endothelium denuded (E-) vessels. **C**, PV3 (10^-6^ g/L) effects on E+ vessels incubated with nitric oxide synthase (NOS) inhibitor L-NAME. The data are reported as means±SE. **P<0.01; ***P<0.0001 (ANOVA and Sidak test).

**Table 1:** Sequence and bioactive potential of the identified peptides present in *Phaseolus vulgaris* (PV3) organized by size, from largest to smallest.

Peptide Sequence	ACEinhibitor	Antioxidant	Dipeptidyl peptidase III inhibitor	Dipeptidyl peptidase IV inhibitor	Stimulant	Antiamnesia	Antithrombotic	Regulators	Activators of ubiquitin mediated proteolysis	Renin inhibitor	CaMPDE inhibitor	Hypolipidemic	Anti inflammatory	Neuropeptide	Opioid
GPWDGLAFVLLPVGSEPKDKGGL	x	x	x	x	x^1,2^	x	x		x						
MYSQDKIIANHIIKVHASAGR	x	x		x	x^1^										
SGPADGLAFVLLPVGSEPKDK	x	x	x	x	x^1,2^	x	x	x^3^	x						
EVHLNKDSTPPSATRVSVV	x	x	x	x											
TTSEALNLANFALNQIL	x		x	x	x^1,2^				x						
TTRSPTFLGLEPAQSTK	x		x	x						x					
SADGIAFALVPVGSQPK	x		x	x	x^1^										
AKIQDKEGIPPDQQR	x			x									x		
SEALNLANFALNQIL	x		x	x	x^1,2^				x						
EALNLANFALNQIL	x		x	x	x^1,2^				x						
SKEGSEYANDAAQR	x		x	x	x^2^					x					
GIAFALVPVGSQPK	x		x	x	x^1^										
TPEAGSAEFTRGS	x		x	x						x	x	x			
QDKEGIPPDQQR	x			x									x		
GPLINPVKSQSS	x			x		x	x	x^3^							
NLANFALNQIL	x		x	x	x^1,2^				x						
TKDYVGDAAQK	x	x	x	x				x^4^							
VSDYADDAAQK	x		x	x				x^4^		x					
APLGQNTVLLR	x	x	x	x	x^1^					x				x	x
MQIFVKTLTG	x			x											
EVEPLPHRK	x	x		x											
NDKEDSVFK	x			x											
EGIPPDQQR	x			x									x		
YVDDAVRR	x		x	x											
TEIPDVRT	*x*	x													
GWDPKQR	x			x											
YEITPDK	x			x											
EIRELR	x	x	x	x						x	x				
ELREIR	x	x	x	x						x					
YDEPVR	x			x											
ELEMR		x	x	x											
EIEMR	x		x	x											
ELELR	x	x		x						x					
ELEIR	x	x		x						x	x				
EIEIR	x	x		x						x	x				

^X^Indicates that the peptide sequence described in the table lines would exert an effect according with columns; ^1^stimulant for glucose uptake; ^2^stimulant to release vasoactive substance; ^3^regulates the activity of the gastric mucous membrane; ^4^ion flow regulators.

As expected, H_2_O_2_ reduced cell viability. PV3 at concentrations of 10 and 20 μg/mL did not cause cell death compared to vehicle group (PV3 10 μg/mL: 96±3% *vs* VEH: 100±1.7%; PV3 20 μg/mL: 96±0.6% *vs* VEH: 100±1.7%; P<0.05). PV3 30 μg/mL increased cell viability (PV3 30 μg/mL: 110±0.4% *vs* 100±1.7%; P<0.05). Cytotoxicity was found only at a concentration of 250 μg/mL, causing 74% cell death, which was greater than that triggered by H_2_O_2_ (PV3 250 μg/mL: 26.7±0.3% *vs* VEH+H_2_O_2_: 50±1.1%; P<0.05). All groups differed from H_2_O_2_ (VEH: 100±1.7% *vs* VEH+H_2_O_2_: 50±1.1%; PV3 10 μg/mL: 96±3% *vs* VEH+H_2_O_2_: 50±1.1%; PV3 20 μg/mL: 96±0.6% *vs* VEH+H_2_O_2_: 50±1.1%; PV3 30 μg/mL: 110±0.4% *vs* VEH+H_2_O_2_ 50±1.1%; PV3 250 μg/mL: 26.7±0.3% *vs* VEH+H_2_O_2_: 50±1.1%; P<0.05) (Figure 1B).

The protective capacity of PV3 (preincubation) in endothelial cells exposed to 3% (v/v) H_2_O_2_-induced oxidative stress for 1 h was evaluated. This pretreatment with PV3 was able to display a protective potential at a concentration of 10 μg/mL (H_2_O_2_+PV3 10 μg/mL: 57.1±2% *vs* H_2_O_2_+VEH: 50.2±1.1%; P<0.05). Concentrations of 20 and 30 μg/mL showed similar cell viability to the vehicle (H_2_O_2_+PV3 20 μg/mL: 47.9±0.3% *vs* H_2_O_2_+VEH: 50±1.1%; H_2_O_2_+PV3 30 μg/mL: 48.1±2.4% vs H_2_O_2_+VEH: 50.2±1.1%; P<0.05). In contrast, the only cytotoxic concentration was 250 μg/mL, in which cell death was increased by 45% (H_2_O_2_+PV3 250 μg/mL: 27.6±1% *vs* VEH+H_2_O_2_: 50.2±.1.1%; P<0.05) (Figure 1C).

The ability of the extract to increase NO release in endothelial cells was evaluated and Figure 2 shows these results obtained by confocal microscopy using the DAF-FM probe. In order to evaluate the PV3 effect on NO production in human endothelial cells, the PV3 concentration chosen was 10 μg/mL, i.e., that capable of exerting protection against oxidative stress (PV3 10 μg/mL: 113.7±2% *vs* VEH+H_2_O_2_:100±2.0%; P<0.05) without evoking cytotoxicity (PV3 10 μg/mL: 96±3% *vs* VEH: 100±1.7%; P<0.05). All treatments differed from vehicle and from each other within this assay. H_2_O_2_ increased NO, but the magnitude of this oxidonitrergic release was even greater when cells were incubated with PV3. Interestingly, the concomitant cell exposure to H_2_O_2_ and PV3 evoked NO release, but these levels were lower than those obtained only with PV3 (VEH: 1.2±0.2%; VEH+H_2_O_2_: 8.2±0.7%; PV3 10 µg/mL: 19.2±1.1%; PV3+H_2_O_2_: 14.7±0.7%; P<0.05 among groups) (Figure 2A and B).

The PV3 influence upon ROS was evaluated by DHE probe and confocal microscopy, shown in Figure 3. PV3 (10 μg/mL) displayed a cytoprotective effect and was the concentration of choice. As expected, ROS was increased when the cells were exposed to H_2_O_2_ for 1 h (VEH: 8.5±1.3% *vs* VEH+H_2_O_2_: 25±1.1%; P<0.05). ROS levels were lower in cells that underwent concomitant treatment with PV3 10 μg/mL (VEH: 8.5±1.3% *vs* PV3 10 μg/mL: 3.6±0.2%; P<0.05). The previous (24 h) incubation of cells with PV3 attenuated the ROS increases evoked by H_2_O_2_ (VEH+H_2_O_2_: 25±1.1% *vs* PV3+H_2_O_2_: 13±0.5%; P<0.05).

PV3 induced a significant relaxation at 10^-6^ and 10^-5^, but not at 10^-7^ g/L concentration in E+ vessels (Figure 4A). The vasorelaxation induced by PV3 10^-6^ g/L was completely blunted in E- aortic rings (Figure 4B), thus indicating that PV3 effects are strongly dependent on endothelium. Endothelium-dependent vasorelaxation effects evoked by PV3 in isolated vessels was also inhibited by the NOS inhibitor L-NAME (Figure 4C).

## Discussion

The method of choice for obtaining PV3 did not use techniques intended for chemical or physical digestion of proteins, i.e., the 35 peptides identified are naturally occurring in the grains. The extraction method we employed was based on the solubility of the protein content in the sample. By using formic acid with acetonitrile solution, the solvation layer and the isoelectric point of the proteins are simultaneously changed. The addition of acetonitrile decreases the dielectric constant of the aqueous fraction in the extracting solution, which changes the polarity of the extract and results in an increased protein solubility with low polarity (18). This method is effective for extraction of amphipathic proteins and peptides. The peptide extract generated from hardened bean residues with low commercial value was tested for potential on cytotoxicity. Subsequently, the PV3 effects on NO release and ROS production were evaluated. Our findings also showed that PV3 displayed little cytotoxicity whereas it reduced ROS production and increased NO release in human endothelial cells and in arteries.

The results on the antioxidant potential by DPPH assay are in agreement with the antioxidant effect of bean peptides previously reported. One prior study showed antioxidant activity by reducing the DPPH radical in proteins isolated from common bean (19). Other authors obtained higher IC50 in DPPH essays: 153.2, 171.89, and 195.95 µg/mL in alkalase, trypsin, and flavorzyne hydrolysates, respectively (20). Following on from this previous evidence, we suggest that the antioxidant activity we found may rely on the aminoacidic composition, since the presence of aromatic amino acid residues (such as tryptophan, tyrosine, and phenylalanine) and of basic amino acids (such as histidine and arginine) may underlie antioxidant activity (21). This shall be supported by the presence of these aforementioned aminoacidic compounds among those detected in our LCMS assays.

Among the 35 peptides identified in the PV3 extract (Table 1), four (PV3-A6, PV3-A7, PV3-A8, and PV3-A9) showed a degree of alignment with a leucoagglutinating phytohemagglutinin (PHA-L) with allergenic potential in BALB/C mice (22). The peptides aligned to this protein had low molecular weight (>1.7 kDa), while the purified PHA-L protein has a molecular weight of 29.5 kDa (22). It is possible that biochemical events related to bean hardening have degraded the protein into smaller peptides. These fragments (i.e., the peptide content found in PV3) may display a structural conformation that would be insufficient for being allergenic *in vivo*. With the presence of these fragments in PV3 arises the necessity for isolation of these fragments and additional tests for assessing the allergenic potential of PV3 should be conducted.

The BIOPEP software analyses showed that all peptides identified in PV3 are potentially able to inhibit ACE, a dipeptidylcarboxypeptidase that converts angiotensin I into the potent vasoconstrictor peptide angiotensin II. In addition, ACE is able to degrade bradykinin (Bk), which is another pressor mechanism, since this Bk degradation reduces the vasodilator effects that would be exerted by binding to Bk receptors (23 [Bibr B24] [Bibr B25]). In this regard, ACE inhibition plays an important role in regulating blood pressure, which explains a worldwide pharmacotherapeutic choice in the management of hypertension and other cardiovascular diseases (23). Many studies report bioactive peptides derived from bean proteins with potential for the treatment of hypertension, due to its ability to inhibit ACE activity (21,). The study of Tagliazucchi et al. (26) identified that the peptide fraction derived from *Phaseolus vulgaris* (also comprising molecules <3 kDa) displays potent ACE inhibitory activity *in vitro.* This fraction, however, was obtained following heating and enzymatic procedures. Contrastingly, the extraction method adopted in our study raises the possibility that the naturally occurring peptides present in PV3 would exert cardiovascular effects by releasing NO, a potent endothelium-derived relaxing factor that counteracts angiotensin II constricting effects (27). This was confirmed by our assays in isolated aortic rings (*ex vivo*), which showed endothelium- and NO-dependent vasodilating effects exerted by PV3. Therefore, further studies should be conducted to verify the PV3 potential hypotensive/antihypertensive effects *in vivo*.

It is noteworthy from current bioinformatics analyses that almost all peptides present in PV3 extract may display a DPP-IV inhibitory potential. DPP-IV is a metabolic enzyme that can degrade and inactivate the glucagon-like peptide-1 from incretins and the glucose-inhibiting polypeptide (28). These incretins induce an increase in insulin secretion (insulinotropic activity) during the postprandial period in response to food intake (28). DPP-IV inhibition has been shown to improve glycemic regulation in type 2 diabetics (28). Several publications have also shown that DPP-IV can be inhibited *in vitro* and in small animals after ingesting hydrolysates and peptides derived from food proteins (29,30). Our pioneer results identifying peptides with potential DPP-IV inhibitory activity raise the need for addressing PV3 effects upon *in vivo* glucose metabolism.

The MTT assay showed that PV3 at concentrations of 10 and 20 μg/mL did not cause cell death. Also, treatment with the concentration of 30 μg/mL increased cell viability. Some peptides (PV3-A10 to PV3-A16) have aligned the sequence with a phytohemagglutinin (PHA), known for inducing leukocyte mitosis (31). In this regard, it is possible that fragments of this mitosis-inducing protein would be (at least partially) in charge of this increased cell viability. Contrary to these advantageous effects found at low concentrations, 76% of cell death was observed when the concentration of PV3 was 250 μg/mL. Similar cytotoxic effects of high concentrations on endothelial cells were previously reported for several compounds that usually display therapeutic effects at low concentration, which include statins, thiazolidinediones, and even insulin (32,33). On the other hand, when PV3 treatment was given before inducing oxidative stress, there was an attenuation on the oxidative effects on cell death levels compared with those detected following H_2_O_2_ exposure, and this may involve antioxidant effects (34).

PV3 increased NO and reduced ROS levels without causing cell death. In addition, PV3 protected endothelial cells from death by H_2_O_2_-induced oxidative stress through ROS reducing mechanisms, which suggests a PV3 antioxidant effect. However, cells treated with a combination of PV3 and H_2_O_2_ showed a reduction in NO compared to the group treated with PV3 alone. One possible explanation for this result may be the complex composition of peptides present in the extract. Similar effects occur with resveratrol, which at low concentrations promotes antioxidant effects, whereas at higher concentrations, it may have a dose-dependent pro-oxidant role likely followed by cell damage and apoptosis (35). In addition, it was possible to observe cells with conserved morphology when treated with 10 μg/mL PV3 in relation to cells treated only with vehicle or H_2_O_2_. This phenomenon persisted even when PV3-treated cells were exposed to H_2_O_2_. It was also observed that PV3-treated cells had higher NO concentration/deposition in the nucleus whereas PV3+H_2_O_2_-treated cells had higher cytoplasmatic NO concentration/deposition. Endothelial dysfunction is known to occur due to decreases in NO bioavailability and increases in endothelin-1 (ET-1), angiotensin II, and oxidant levels, which contribute to an imbalance in endothelium-derived relaxing and constricting factors (36). Thus, it is not implausible to propose that PV3 could improve endothelial function in diseases such as arterial hypertension. This hypothesis is further strengthened by the evidence from our *ex vivo* assays: PV3 vasorelaxant actions rely on endothelial-dependent oxidonitrergic paths. Since increases in NO availability would also modify platelet aggregation (37), it is worth suggesting concomitant beneficial endothelial and platelet effects of PV3.

Epidemiological studies show that intake of whole grains is correlated with a reduction in the incidence of cardiovascular diseases, diabetes, and cancer (38,39). The whole grain phytochemicals that exert antioxidant actions may attenuate ROS contribution to the etiology and to the pathophysiology of these diseases (27), thus resulting in benefits for human health (40). Although there is still a need for further (*in vivo*) studies, our data on the induction of NO release and on the reduction of oxidative stress induced by PV3 let us to propose that the current peptide extract would be able to increase NO bioavailability concomitantly to producing an antioxidant effect and this may be reached even by the consumption of hardened bean as a nutraceutical source.

We concluded that the low molecular weight peptide extract produced from bean residues with no commercial value displayed low cytotoxicity, reduced ROS, and increased NO in endothelium. This study suggests the PV3 potential therapeutic use to improve NO-dependent physiological functions and to reduce oxidative stress.

## Supplementary Material

Click here to view [pdf].
